# Altered Coupling of Psychological Relaxation and Regional Volume of Brain Reward Areas in Multiple Sclerosis

**DOI:** 10.3389/fneur.2020.568850

**Published:** 2020-10-06

**Authors:** Katharina Wakonig, Fabian Eitel, Kerstin Ritter, Stefan Hetzer, Tanja Schmitz-Hübsch, Judith Bellmann-Strobl, John-Dylan Haynes, Alexander U. Brandt, Stefan M. Gold, Friedemann Paul, Martin Weygandt

**Affiliations:** ^1^Charité – Universitätsmedizin Berlin, Corporate Member of Freie Universität Berlin, Humboldt-Universität zu Berlin, and Berlin Institute of Health, NeuroCure Clinical Research Center, Berlin, Germany; ^2^Charité – Universitätsmedizin Berlin, Corporate Member of Freie Universität Berlin, Humboldt-Universität zu Berlin, Berlin Institute of Health (BIH), Department of Psychiatry and Psychotherapy, Berlin, Germany; ^3^Charité – Universitätsmedizin Berlin, Corporate Member of Freie Universität Berlin, Humboldt-Universität zu Berlin, and Berlin Institute of Health, Berlin Center for Advanced Neuroimaging, Berlin, Germany; ^4^Max Delbrück Center for Molecular Medicine and Charité – Universitätsmedizin Berlin, Corporate Member of Freie Universität Berlin, Humboldt-Universität zu Berlin, and Berlin Institute of Health, Experimental and Clinical Research Center, Berlin, Germany; ^5^Charité – Universitätsmedizin Berlin, Corporate Member of Freie Universität Berlin, Humboldt-Universität zu Berlin, Berlin Institute of Health, Bernstein Center for Computational Neuroscience, Berlin, Germany; ^6^Department of Neurology, University of California, Irvine, CA, United States; ^7^Institute of Neuroimmunology and Multiple Sclerosis (INIMS), University Medical Center Hamburg Eppendorf, Hamburg, Germany; ^8^Charité – Universitätsmedizin Berlin, Corporate Member of Freie Universität Berlin, Humboldt-Universität zu Berlin, and Berlin Institute of Health, Department of Psychiatry and Psychotherapy, Berlin, Germany; ^9^Charité – Universitätsmedizin Berlin, Corporate Member of Freie Universität Berlin, Humboldt-Universität zu Berlin, and Berlin Institute of Health, Department of Psychosomatic Medicine, Berlin, Germany; ^10^Charité – Universitätsmedizin Berlin, Corporate Member of Freie Universität Berlin, Humboldt-Universität zu Berlin, and Berlin Institute of Health, Department of Neurology, Berlin, Germany

**Keywords:** multiple sclerosis, autoimmunity, neuroinflammation, gray matter, ventromedial prefrontal cortex (vmPFC), psychological relaxation, psychophysiological stress responses, brain reward system

## Abstract

**Background:** Psychological stress can influence the severity of multiple sclerosis (MS), but little is known about neurobiological factors potentially counteracting these effects.

**Objective:** To identify gray matter (GM) brain regions related to relaxation after stress exposure in persons with MS (PwMS).

**Methods:** 36 PwMS and 21 healthy controls (HCs) reported their feeling of relaxation during a mild stress task. These markers were related to regional GM volumes, heart rate, and depressive symptoms.

**Results:** Relaxation was differentially linked to heart rate in both groups (*t* = 2.20, *p* = 0.017), i.e., both markers were only related in HCs. Relaxation was positively linked to depressive symptoms across all participants (*t* = 1.99, *p* = 0.045) although this link differed weakly between groups (*t* = 1.62, *p* = 0.108). Primarily, the volume in medial temporal gyrus was negatively linked to relaxation in PwMS (*t* = −5.55, p_family−wise−error(FWE)corrected_ = 0.018). A group-specific coupling of relaxation and GM volume was found in ventromedial prefrontal cortex (VMPFC) (*t* = −4.89, p_FWE_ = 0.039).

**Conclusion:** PwMS appear unable to integrate peripheral stress signals into their perception of relaxation. Together with the group-specific coupling of relaxation and VMPFC volume, a key area of the brain reward system for valuation of affectively relevant stimuli, this finding suggests a clinically relevant misinterpretation of stress-related affective stimuli in MS.

## Introduction

Multiple sclerosis (MS) is an inflammatory disease of the central nervous system of presumed autoimmune origin. It manifests most frequently in young female adults and leads to demyelination, neurodegeneration, and axonal damage (1, 2).

Beyond key neurological symptoms, persons with MS (PwMS) are characterized by stress-related syndromes such as depression and anxiety which strongly affect social and work-related factors (3). Adult PwMS score higher on questionnaires assessing childhood traumatization than controls (4), and the occurrence of stressful life-events is coupled to subsequent exacerbation of neurologic symptoms (5). Neuroendocrine studies identified altered functioning of biological stress systems in PwMS [e.g., hypercortisolism for the hypothalamo-pituitary-adrenal axis (HPA); (6)] and reduced levels of norepinephrine in peripheral blood lymphocytes for the sympathetic nervous system (7) and altered immuno-modulatory effects of these systems [e.g., reduced corticoid-induced immunosuppression; (8)]. Structural neuroimaging studies showed that HPA hyperactivity is positively linked to atrophy of hippocampal subregions (9) and ventricular size (10) in PwMS. Finally, using a mild cognitive functional MRI (fMRI) stress task, we showed that blunted stress responses in insular cortex are linked to stronger clinical disability in PwMS (11). Together, these findings suggest an important role of stress for MS and thus advocate for a more thorough understanding of factors potentially counteracting its effects.

The ability to relax during stress exposition or therapeutic relaxation techniques might be such factors. First, the ability to relax is an important aspect of resilience, a psychological stress adaption mechanism (12) that is accompanied by higher quality of life (QoL) and lower anxiety and depression in PwMS (13). Second, therapeutic relaxation induced by autogenic training improves health-related QoL in PwMS (14) and, within a larger stress management program, contributed to a reduction of the incidence of novel lesions in a randomized controlled trial (15).

Despite these findings underlining the importance of relaxation for counterbalancing stress effects in PwMS, however, nothing is currently known about neurobiological factors underlying relaxation in PwMS. For this reason, and given the previously shown link between stress-system hyperactivity and regional gray matter (GM) atrophy in PwMS (9), we used a mild cognitive stress task comprising mental arithmetic and performance feedback to measure the level of perceived relaxation during different levels of stress exposition in 36 PwMS and 21 healthy controls (HCs). Perceived stress was additionally measured to evaluate the utility of the task to induce a stress response and to clarify the relation of task-induced relaxation and stress. Perceived relaxation was then related to the participants' regional GM volumes, task-induced heart rate variations, and degree of depressive symptoms to provide insights into factors underlying relaxation in MS. Driven by findings of an “impaired emotional reactivity” or impaired ability to detect and process emotional threat cues in PwMS and HCs, respectively (16), we tested two main hypotheses, i.e., whether relaxation is differentially related to (1) the regional volume of brain areas involved in affective processes and (2) to stress-related heart-rate accelerations in PwMS vs. HCs.

## Methods

### Participants

Seventy individuals (43 PwMS and 27 HCs) were screened for participation in this study. PwMS were recruited in cooperation with the Charité neuroimmunology outpatient clinic whereas HCs were enrolled by advertisements. To be included, patients had (1) to be diagnosed with relapsing-remitting (RRMS) or secondary progressive MS (SPMS) according to the McDonald Criteria of 2010 (17), (2) be without disease-modifying treatment or have a stable treatment for at least 6 months, (3) be at least 18 years old, and (4) be able to participate in the stress paradigm without physical or mental restriction. Exclusion criteria were acute MS relapses and unstable MS courses, other neurological disorders, an existing diagnosis of a psychiatric disorder, and MRI contraindications. If applicable, inclusion and exclusion criteria were identical for HCs. After applying these criteria, 57 participants were included [35 females; 36 MS patients (27 RRMS, 9 SPMS)]. Written informed consent was obtained from participants according to the Declaration of Helsinki (18). The study was approved by the research ethics committee of the Charité—Universitätsmedizin Berlin (EA1/182/10, amendment V).

Rating data, structural MRI, and data on the extent of depressive symptoms were available for all 57 participants, pulse data for a subset of 46 participants. Structural MRI data were also evaluated for different purposes in (11, 19, 20). Furthermore, pulse data, and stress but not relaxation ratings were also evaluated in (11).

### Clinical Assessment

One experienced neurologist examined all MS patients by conducting the Expanded Disability Status scale (EDSS) (21) to assess clinical disability. PwMS and HCs completed the Beck Depression Inventory II (22). For further parameters, please see (19, 20).

### Experimental Task, Psychophysiological Stress, and Relaxation Measures

The stress paradigm comprised seven stages and had a total duration of 44 min. In the first stage (“Rating I”), participants rated their current level of feeling relaxed, stressed, anxious, and frustrated on a 9-level Likert scale (ranging from “not at all” to “very strong”). The selection of a given intensity for each psychological marker was performed with an MRI-compatible response button box. In stage 2, resting fMRI [for an analysis of fMRI data, see (11)] and pulse data were measured. In stage 3 (“Rating II”), which took place immediately after fMRI, the participants again provided self-report data for the four psychological measures. In stage 4, the mental arithmetic stress task was conducted while participants were scanned with fMRI and pulse data were assessed (“Stress task”). The task was derived from the Trier Social Stress Test (23) and had a total duration of 12 min. In the paradigm, the participants had to perform a series of subtraction tasks (i.e., “operand X minus operand Y”) as fast as possible. The two operands were depicted in the middle of a computer screen inside the scanner. To solve a task, the participants had to select the single correct result from one of four numbers depicted on the bottom of the screen with the button box. Across the first 10 correct trials or maximally during the trials of the first 4 min of the task (i.e., during the “evaluation stage”), a given participant's performance level (i.e., the average time necessary to find the correct solution) was evaluated to provide feedback in the remaining period of the 12 min (i.e., during the “performance stage”). Specifically, depending on the difference between the time necessary to find a correct solution in trials of the performance stage and the participant's average performance in the evaluation stage, each trial's performance was evaluated in terms of school grades ranging from “1—sehr gut” (very good) to “5—ungenügend” (insufficient). Moreover, the time provided to solve each task was adopted based on the participant's performance in the performance stage: If the preceding trial was correctly solved, the time provided was reduced by 10% in the following trial. Contrarily, it was prolonged by 10% if the answer was false. This temporal adaptation mechanism was employed to guarantee that all participants constantly performed at their maximal personal ability level. In the fifth stage, the participants provided self-report data with regard to the four psychological scales immediately after fMRI scanning (“Rating III”). In stage 6, a post-stress resting fMRI and pulse measurement was conducted. Finally, the stress paradigm ended after the participants provided rating data for the four scales for a fourth time (“Rating IV”) in the seventh stage.

**Figure 1:**
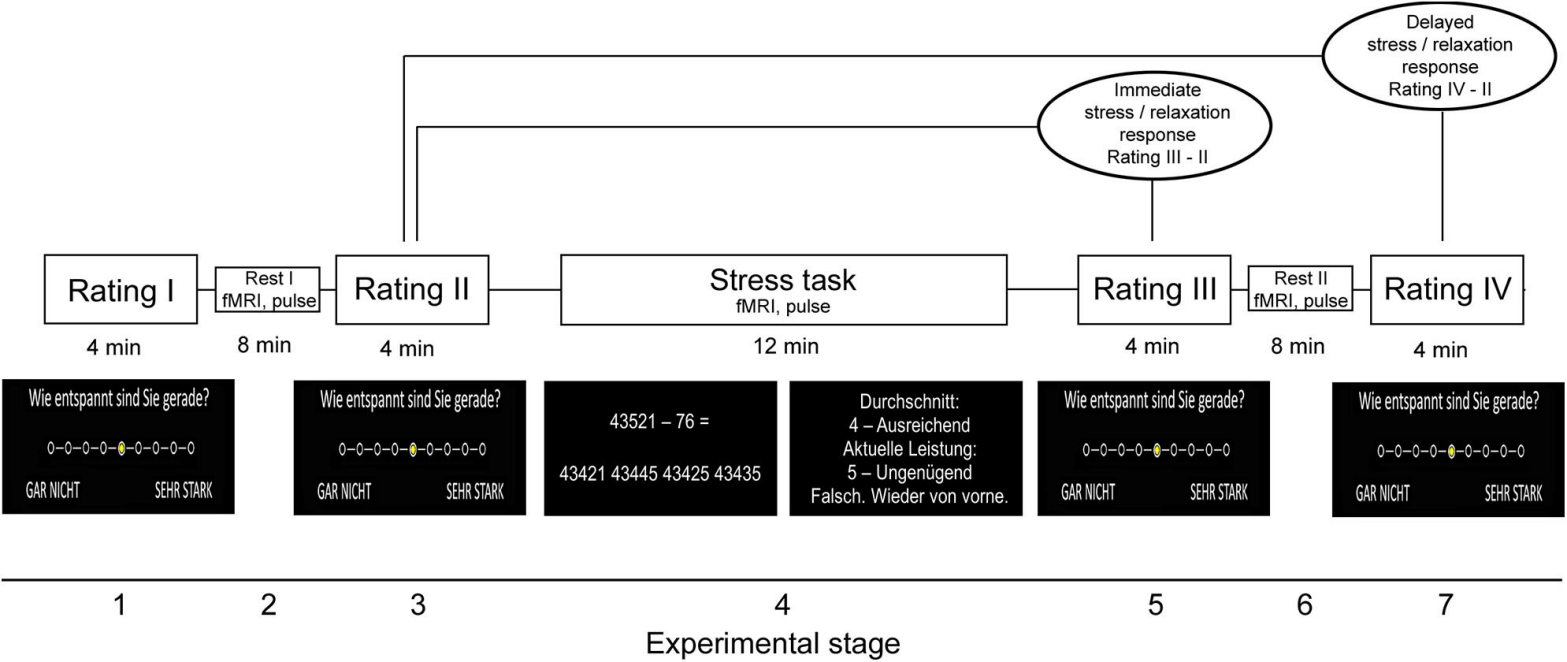
Stress paradigm. Please note that the paradigm initially also included a salivary cortisol measurement during all four rating periods. Because we do not refer to these data in this study, we would like to point the reader to (11) from which also parts of the figure were taken.

Given that stress and thus potentially relaxation responses differ in terms of immediate and delayed or sustained responses (11, 24), we computed the differences between relaxation reported in the post-stress (“Rating III”) minus in the pre-stress stage (“Rating II”) as immediate and relaxation reported in the seventh stage (“Rating IV”) minus ratings obtained in the pre-stress stage (“Rating II”) as delayed relaxation measure. The corresponding markers were also computed for perceived stress. Differences in the average heart rate during the last 8 min of (see next section) fourth minus the second stage were computed as immediate peripheral stress response markers, differences between stages 6 and 2 as markers of delayed peripheral stress responses. Finally, we computed the task load for each participant as the average duration of the inter-trial interval during the final 8 min of the stress task. We focused on the final 8 min for heart rate and performance computation to ensure equal feedback settings across participants. Figure 1 illustrates the paradigm.

### Heart Rate Measurement

Pulse data were assessed during functional imaging with a standard pulse oximeter (included in the Physiological Monitoring Unit (PMU) of the MR scanner; Magnetom Trio, Siemens, Erlangen, Germany) which measures variations in infrared light transmission (50 Hz sampling frequency). To avoid transmitter dislocation potentially caused by button presses during the task, the pulse oximeter was placed on the participants' toes. The quality of pulse signals assessed with this measurement approach has been proven to be comparable with that of signals assessed at participants' fingers (25). We filtered the heartbeat signal for denoising and thus excluded (1) heartbeats inducing an acceleration to 133% or higher or a deceleration to 75% or below and (2) signals within the baseline oximeter raw signal (i.e., raw signal values below 2000). To ensure equal feedback settings across participants (see task description), we only evaluated heart rate signals measured during the final 8 min of the stress task.

### MRI Sequences

Structural brain images were measured using a 3-T whole-body tomograph (Magnetom Trio; Siemens) with a standard head coil (12 channels). Specifically, anatomical T1-weighted (T1w) images were measured with a 3D-magnetization prepared rapid gradient echo MP-RAGE sequence using these parameters: 176 slices; slice thickness 1.3 mm; in-plane voxel resolution 1.5 · 1.5 mm^2^; TR = 1,720 ms; TE = 2.34 ms; FA = 9°; FOV = 192 · 192 mm^2^; matrix size = 128 × 128; duration 1 min and 43 s. Moreover, sagittal T2-weighted (T2w) images were acquired to facilitate manual lesion mapping with these parameters: 176 slices; 1-mm isotropic voxels; TR = 5,000 ms; TE = 502 ms; FA = 120°; FOV = 256 · 256 mm^2^; matrix size = 256 · 256; 5 min and 52 s duration.

### MRI Preprocessing

Preprocessing of anatomical MRI images comprised four larger steps, i.e., (1) a manual lesion mapping, (2) the determination of tissue probability voxel maps, (3) determination of a gray matter group mask, and (4) a determination of the intracranial volume.

#### (i) Lesion Mapping

To determine participant-specific lesion masks, an expert rater from the group of Friedemann Paul performed a manual lesion mapping procedure based on high-resolution T2w-images with the OsiriX software toolbox (OsiriX Foundation, Geneva, Switzerland). The lesion masks were exactly the same as the ones used in (11).

#### (ii) Determination of Tissue Probability Voxel Maps

To compute voxel maps assessing the probability with which each voxel in the brain belongs to GM, white matter (WM), or cerebrospinal fluid (CSF), we first used SPM12 to co-register the T2w images and the lesion masks derived from the T2w images to the T1w anatomical images. In the next step, we spatially registered/normalized the T1w anatomical image and the co-registered lesion mask of each patient to the anatomical standard space defined by the Montreal Neurological Institute (MNI) (26) brain template. For this step, we used the combined spatial normalization and segmentation algorithm included in SPM12. The algorithm simultaneously computes the spatial registration/transformation as well as voxel-wise tissue probability maps for GM, WM, and CSF in the original subject-specific and the anatomical standard space. In particular, the algorithm determines modulated tissue probability maps in the anatomical standard space that consider/control for the impact of spatial deformations applied during the spatial normalization which might vary between PwMS and HCs due to MS-induced atrophy. In addition, the algorithm also computes non-modulated tissue probability maps which were however not used in the present study. Coordinates located in lesioned tissue as reflected by the co-registered lesion masks of a subject were not used for this registration procedure. The voxel size of modulated tissue probability images determined in the anatomical standard space was 3 · 3 · 3 mm^3^. The (all) tissue probability maps computed were the same as the ones used in (11). The tissue probability voxel images reflecting the modulated probability with which voxels belong to GM were entered in the MRI group analyses.

#### (iii) Determination of a GM Group Mask

To constrain all MRI group analyses to brain areas located in GM, we then determined a group mask for this tissue across all participants in three steps. In particular, we computed the voxel-wise average tissue probability (based on modulated and non-smoothed tissue probability maps determined in the anatomical standard space; see previous section) for GM, WM, and CSF across all 57 participants in the first step. Afterwards, a voxel coordinate was assigned to that tissue for which the maximal averaged tissue probability was determined. Voxel coordinates for which GM had the maximal average tissue probability were included in the GM group mask. In the second step, we removed voxel coordinates from the mask generated in step 1 that were located in lesioned tissue (and the six direct neighbor voxel coordinates having a Euclidean distance of exactly one voxel to control for potential partial voluming effects) in at least one patient as determined by spatially normalized lesion map of a patient to ensure that lesions did not affect our subsequent MRI group analyses. Finally, in the third step, we removed coordinates located outside the mask of the intracranial volume provided by SPM12. Following these three steps, the coordinates included in our GM group mask spanned 46% of the total intracranial volume.

#### (iv) Determination of the Intracranial Volume

The participant-specific intracranial volume (ICV) was computed as the number of voxels with non-zero tissue probability determined across all tissue types and based on tissue probability maps in the original, subject-specific image space and the respective co-registered T2w lesion masks. The ICV was used as covariate of no interest in our MRI group analyses (see Supplementary Material).

### Statistical Analyses of Psychophysiological Relaxation and Stress Measures

This set of analyses comprised four individual analyses. In psychophysiological analysis 1, we evaluated basic effects of task exposure on psychophysiological relaxation and stress response parameters. Specifically, we tested basic effects of task exposure (i.e., task stage) on perceived relaxation, perceived stress, and heart rate variations across all participants independent of group membership (main effect of task stage), whether these measures differed between groups independent of task stage (main effect of group), and finally whether the impact of individual task stages differed between groups (interaction effect of task stage and group). This was separately done for immediate and delayed relaxation, stress, and heart rate in six individual factorial linear-mixed effects (LMEs) analyses [cf. (27)]. Each of these six analyses was done using the FITLMEMATRIX algorithm implemented in Matlab 2014a (MathWorks, Natick, Massachusetts, USA). A regressor coding for the task stage served as first fixed covariate of interest (CI). In particular, this regressors coded zeros for stage 3 and ones for stage 5 for analyses of immediate stress or relaxation and zeros for stage 3 and ones for stage 7 for analyses of delayed stress or relaxation. Similarly, it coded zeros for stage 2 (2) and ones for stage 4 (6) for analyses of immediate (delayed) heart rate variations. The second fixed CI was a regressor for group (0 HC, 1 PwMS), and the third an interaction regressor computed as the product of task stage and group. Regressors for sex, age, and task load (plus constant) were entered as fixed covariates of no interest (CNI), and a constant as random CNI. Ratings obtained in stages 3 and 5 served as criteria in analyses of immediate effects, those obtained during stages 3 and 7 in analyses of delayed effects. Averaged heart rate signals measured during stages 2 and 4 served as criteria for the analysis of immediate heart rate variations, average pulse signals sampled in stages 2 and 6 for delayed pulse variations. To compute measures of inferential statistics, we used permutations testing [5,000 permutations (28)].

In psychophysiological analysis 2, we investigated the association between relaxation and stress as well as between immediate and delayed relaxation. Specifically, we used robust regression (i.e., the ROBUSTFIT Matlab algorithm) to predict the two relaxation difference markers based on the two corresponding stress difference markers across all 57 participants. Sex, age, task load, and a constant served as CNI. In addition, we predicted our delayed relaxation marker based on the marker assessing immediate relaxation.

To test the effects of relaxation, group, and their interaction on peripheral stress responses, we conducted one factorial robust regression analysis to model immediate and one to model delayed heart rate variations in psychophysiological analysis 3. Specifically, regressors for immediate (delayed) relaxation, group, and their interaction (calculated as the product of the given relaxation difference marker and group) served as CI, sex, age, and task load (plus constant) as CNI. Immediate or delayed heart rate variations served as criteria. Permutation testing was used for inference (5,000 permutations).

Finally, in psychophysiological analysis 4, we analyzed the clinical relevance of our relaxation parameters by relating them to depressive symptoms (i.e., BDI-II). These analyses were identical to the ones described for step 3, except for the fact that BDI-II scores and not averaged heart rate served as criterion in all analyses. For all analyses of psychological stress and relaxation, we report the t-statistic for the CI and the false-positive rate for this t-statistic as determined by permutation testing (α = 0.05).

### Statistical Analysis of Structural MRI Data

In voxel-based morphometry (VBM) analyses performed with SnPM13 (https://warwick.ac.uk/fac/sci/statistics/staff/academic-research/nichols/software/snpm), we tested (i) associations of perceived relaxation and regional GM volume in PwMS (i.e., the main effect of relaxation on GM volume in PwMS), and (ii) whether the association between perceived relaxation and regional GM volume differs between PwMS and HCs (i.e., the interaction effect of relaxation and group on regional GM volume). SnPM13 is a toolbox for SPM12 (Wellcome Trust Center for Neuroimaging, Institute of Neurology, UCL, London, UK—http://www.fil.ion.ucl.ac.uk/spm) that uses permutation testing for inference and the maximum statistic approach to control for multiple comparisons. Each of these analyses was once conducted for immediate and once for delayed relaxation. Sex, age, task load, and ICV (plus constant) served as CNI in (i) and (ii), main effect regressors coding for the given relaxation marker and group additionally in (ii). All analyses were constrained to voxel coordinates located in a GM group mask. In each analysis, 5,000 permutations were performed to assess significance. We report coordinates for voxels showing a significant positive or negative effect according to a multiple comparison or family-wise error (FWE) corrected threshold (α_FWE_ = 0.05) in the anatomical standard space defined by the MNI (26). Furthermore, we report the t-statistic for this voxel, the FWE-corrected *p*-value, and the spatial extent/volume of voxels reaching significance. Please note that we report the results of four additional VBM analyses in the Supplementary Material. Specifically, (1) the first one evaluated differences in the regional GM volume between PwMS and HCs, and (2) another one that repeated the two main VBM analyses reported earlier which however were anatomically constrained to those areas identified in the first supplementary analysis. Moreover, (3) a third one investigated associations between relaxation and regional GM volume in HCs only, and (4) the fourth tested the link between psychological relaxation measures and regional GM volume in PwMS but additionally modeled the putative impact of progressive MS type (i.e., RRMS vs. SPMS) as covariate of no interest.

## Results

### Sample Characteristics

The median age in PwMS was 49.7 years (range 21.9–61) and 51.4 years (range 25.1–64.0) in HCs (*t* = −0.59; *p* = 0.547). Twenty-two of 36 PwMS and 13 of 21 HCs were female (χ^2^ = 0.00, p > 0.999). Also, 58.3% of the patients and 76.2% of HCs had a high school diploma (χ^2^ = 1.86, *p* = 0.250). Although none of the PwMS had a clinical diagnosis of a depressive disorder at the time of investigation, depressive symptoms (i.e., BDI-II corrected for effects of sex and age) were higher in PwMS than HCs (PwMS: median: 5.5, range: 0–22; HCs: median: 1, range: 0–10; *t* = 2.98, *p* = 0.005).

Patients showed a median EDSS of 3.5 (range 1.5–6). Fourteen patients were treated with subcutaneous disease-modifying medication (7 with β-interferons, 7 with glatiramer acetate) and 16 with oral medication (8 with dimethylfumarate, 6 with fingolimod, 2 with teriflunomide), while 6 patients were not on any pharmacological treatment. The median disease duration was 6.4 (range 0.3–21.2) years, median number of relapses since diagnosis was 5 (range 1–21), and the median number of days since the last relapse was 654 days (range 22–3,550).

### Statistical Analysis of Psychophysiological Relaxation and Stress Measures

**Figure 2:**
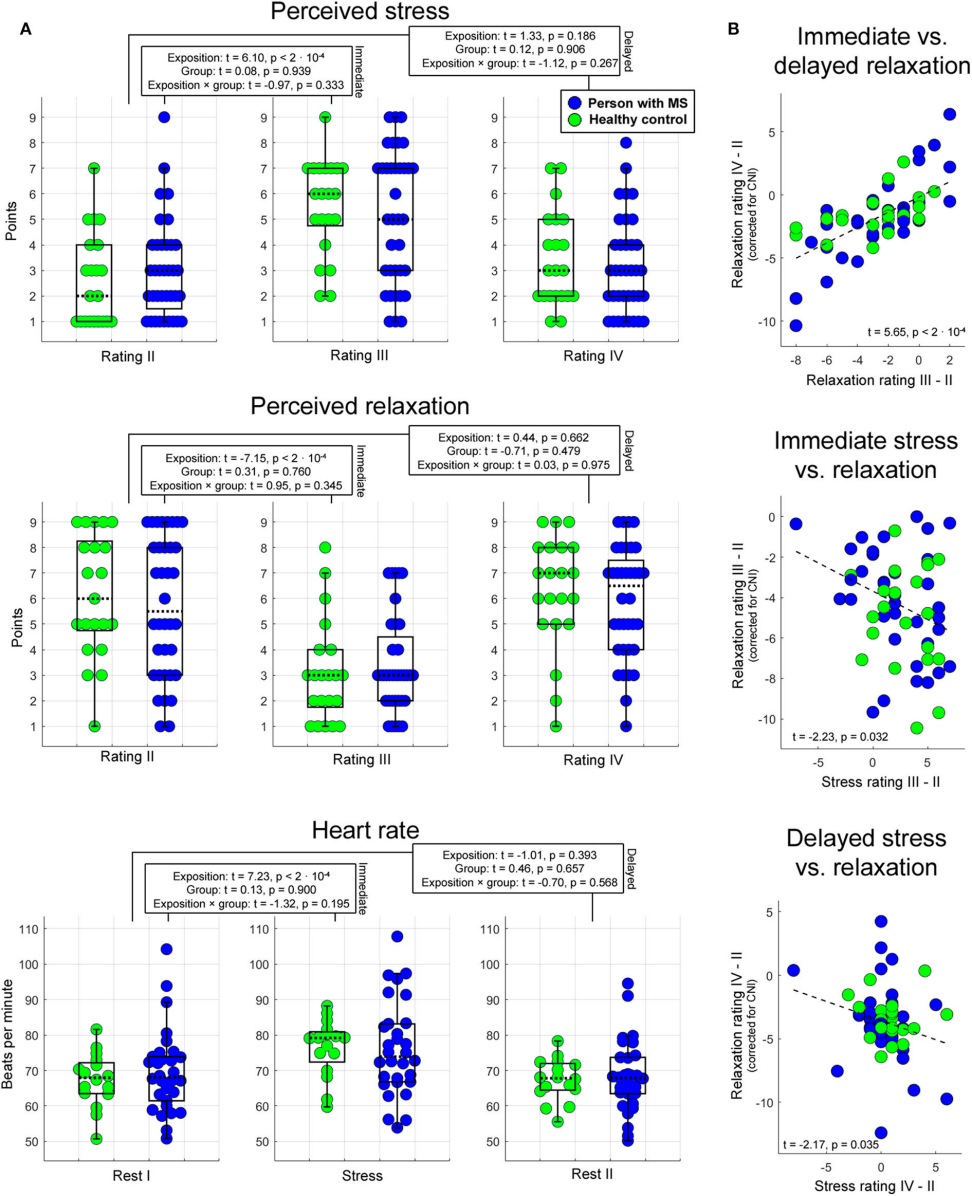
Psychophysiological stress and relaxation responses. Boxplots in **(A)** illustrate the impact of task stage, group, and their interaction on immediate and delayed stress, relaxation, and heart rate. In each boxplot, the dotted line corresponds to the median of a given measure, and the upper (lower) edge of a box to the third (first) quartile of a given measure. Whiskers highlight the most extreme points not considered as outliers. The outlier range was considered to start at the first [third] quartile minus [plus] 1.5 times the interquartile range. As indicated by these graphs, the proportion of outliers was very small. The statistics reported were computed with LME regression (see text for details). Scatterplots in **(B)** show the association between immediate and delayed relaxation, immediate stress and relaxation, and delayed stress and relaxation.

Analyses of basic task effects (psychophysiological analysis 1) showed that the transition from the pre-stress stage (stage 3 for stress and relaxation, stage 2 for heart rate) to the stress task (heart rate: stage 4) or the first post-stress stage, respectively (stage 5 for stress and relaxation) induced a significant variation in all three psychophysiological parameters independent of group membership (main effect of task stage for immediate responses). For delayed effects, no main effect of task stage was found. Groups did not differ in any of the three measures independent of task stage—neither in terms of immediate nor in terms of delayed response (i.e., no main effects of group were found). Finally, task stages also did not exert a different impact on the three measures in the two different groups—neither for immediate nor for delayed responses (i.e., no interaction effects task stage × group were found). These findings are summarized in Figure 2A.

Psychophysiological analysis 2 investigating associations between relaxation and stress as well as between immediate and delayed relaxation showed that (1) immediate relaxation was negatively linked to immediate stress (*t* = −2.23, *p* = 0.032). In this analysis, 8% of the variance in the immediate relaxation marker (corrected for CNI) were explained by the variation in the immediate stress marker. Consistently, (2) a comparable relation was obtained for the corresponding markers assessing delayed effects (*t* = −2.17, *p* = 0.035; 4% explained variance). Finally, the association between immediate and sustained relaxation was strong (*t* = 5.65, *p* < 2 · 10^−4^; 47% explained variance). Findings regarding psychophysiological analysis 2 are shown in Figure 2B.

**Figure 3:**
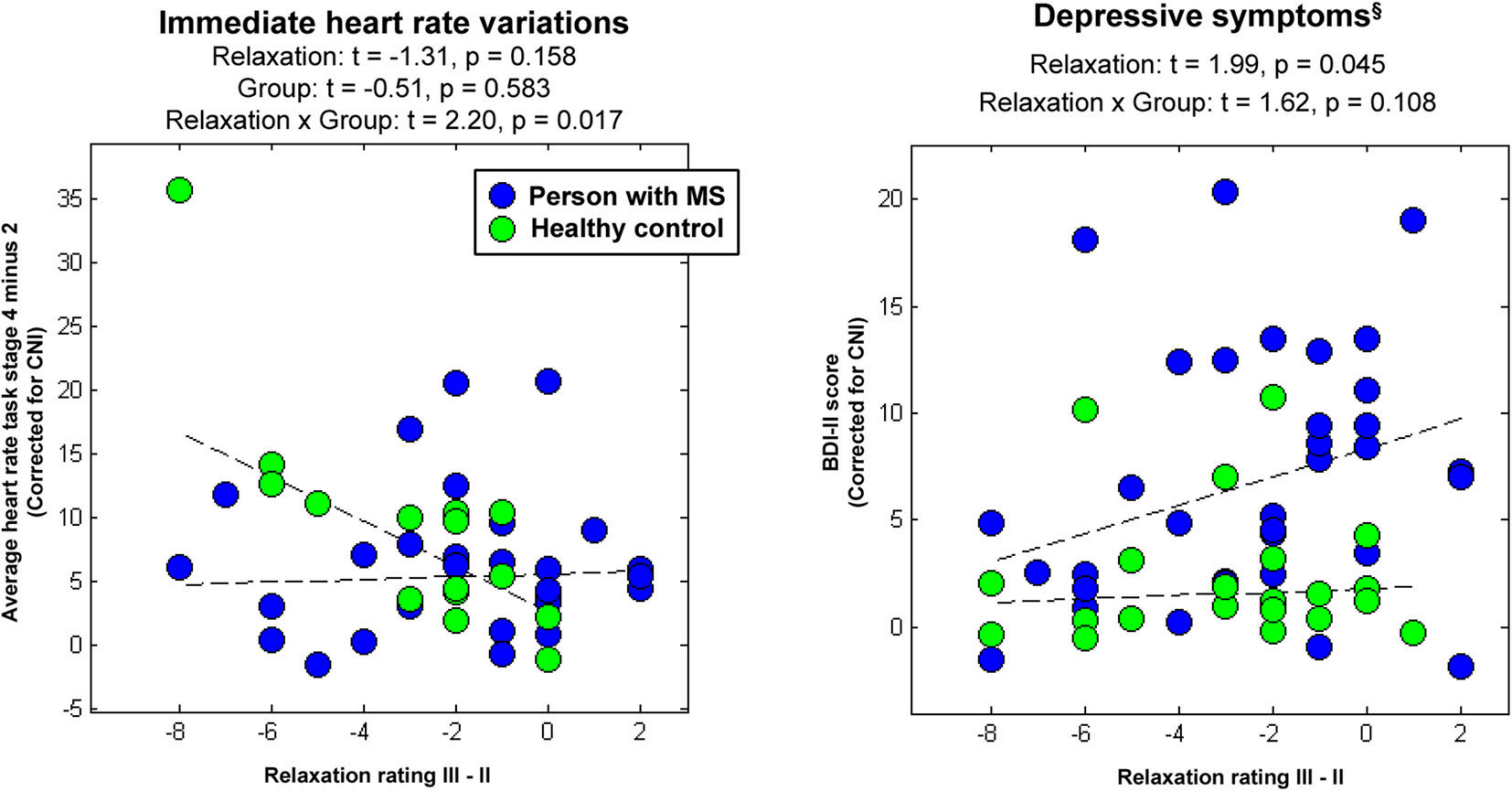
Associations between immediate relaxation and task-induced heart-rate variations or depressive symptoms, respectively. ^§^Please again note that we do not provide the group differences in depressive symptoms computed in the factorial analyses at this place for the same reason as reported in Table 1.

In psychophysiological analysis 3, which evaluated effects of relaxation, group, and their interaction on peripheral stress responses, we found that an (immediate) increase in heart rate from stage 2 (Rest I) to 4 (Stress task) induced a significantly stronger reduction in perceived relaxation in HCs than PwMS. Patients' perception of relaxation apparently did not reflect task-induced immediate variations in heart rate (interaction effect immediate relaxation × group on immediate heart variations; *t* = 2.20, *p* = 0.017; left graph in Figure 3). No interaction effect for delayed responses was found. Groups did not differ in terms of immediate or delayed heart rate responses—neither for immediate nor for delayed responses (i.e., no main effect of group was found) and no overall association of relaxation and heart rate variations was found—neither for immediate nor for delayed responses (i.e., no main effect of relaxation was found). Table 1A shows the statistics for all reported effects evaluated in psychophysiological analysis 3.

Table 1**(A)** Relation of relaxation and heart rate as well as **(B)** relation of relaxation and depressive symptoms.

**Table:** 

**(A) Relaxation, group, and heart rate**			
**Predictor/criterion**	**Relaxation (immediate)**	**Group**	**Relaxation (immediate) × group**
Pulse (immediate)	*t* = −1.31 *p* = 0.156	*t* = −0.51 *p* = 0.594	*t* = 2.20 *p* = 0.017
	**Relaxation (delayed)**	**Group**	**Relaxation (delayed) × group**
Pulse (delayed)	*t* = 0.18 *p* = 0.856	*t* = 0.12 *p* = 0.903	*t* = 0.14 *p* = 0.887

**Table:** 

**(B) Relaxation, group, and depressive symptoms^§^**				
**Predictor/criterion**	**Relaxation (immediate)**	**Relaxation (immediate) × group**	**Relaxation (delayed)**	**Relaxation (delayed) × group**
BDI-II	*t* = 1.99 *p* = 0.045	*t* = 1.62 *p* = 0.108	*t* = 0.89 *p* = 0.380	*t* = 0.76 *p* = 0.449

§ *Please note that we do not report the group differences in depressive symptoms computed in the factorial analyses at this place because this would necessitate reporting two varying differences (due to the varying covariation among predictors)—one computed for the model with immediate and one for the model with delayed relaxation as covariate of interest. Instead, we consider the difference reported in the Results section Sample characteristics (including sex and age as covariates of no interest) as reference (i.e., t = 2.98, p = 0.005)*.

Finally, in psychophysiological analysis 4, which evaluated effects of relaxation, group, and their interaction on the extent of depressive symptoms (i.e., BDI-II), we found an association of immediate relaxation and BDI-II scores across all participants (main effect of immediate relaxation; *t* = 1.99, *p* = 0.045; right graph in Figure 3). Table 1B shows the statistics for all effects evaluated in psychophysiological analysis 4.

### Statistical Analysis of Structural MRI Data

#### Associations of Perceived Relaxation and Regional GM Volume in PwMS

In the first VBM analysis testing main effects of relaxation on regional GM volume in PwMS, we found a negative association between immediate relaxation in fusiform gyrus (FG) and middle temporal gyrus (MTG; in other words, higher GM volume in these areas was linked to lower immediate relaxation). For delayed relaxation and GM, a negative link was found for volume in the MTG and enthorinal cortex (ENTH).

#### Differential Association of Perceived Relaxation and Regional GM Volume in PwMS and HCs

**Figure 4:**
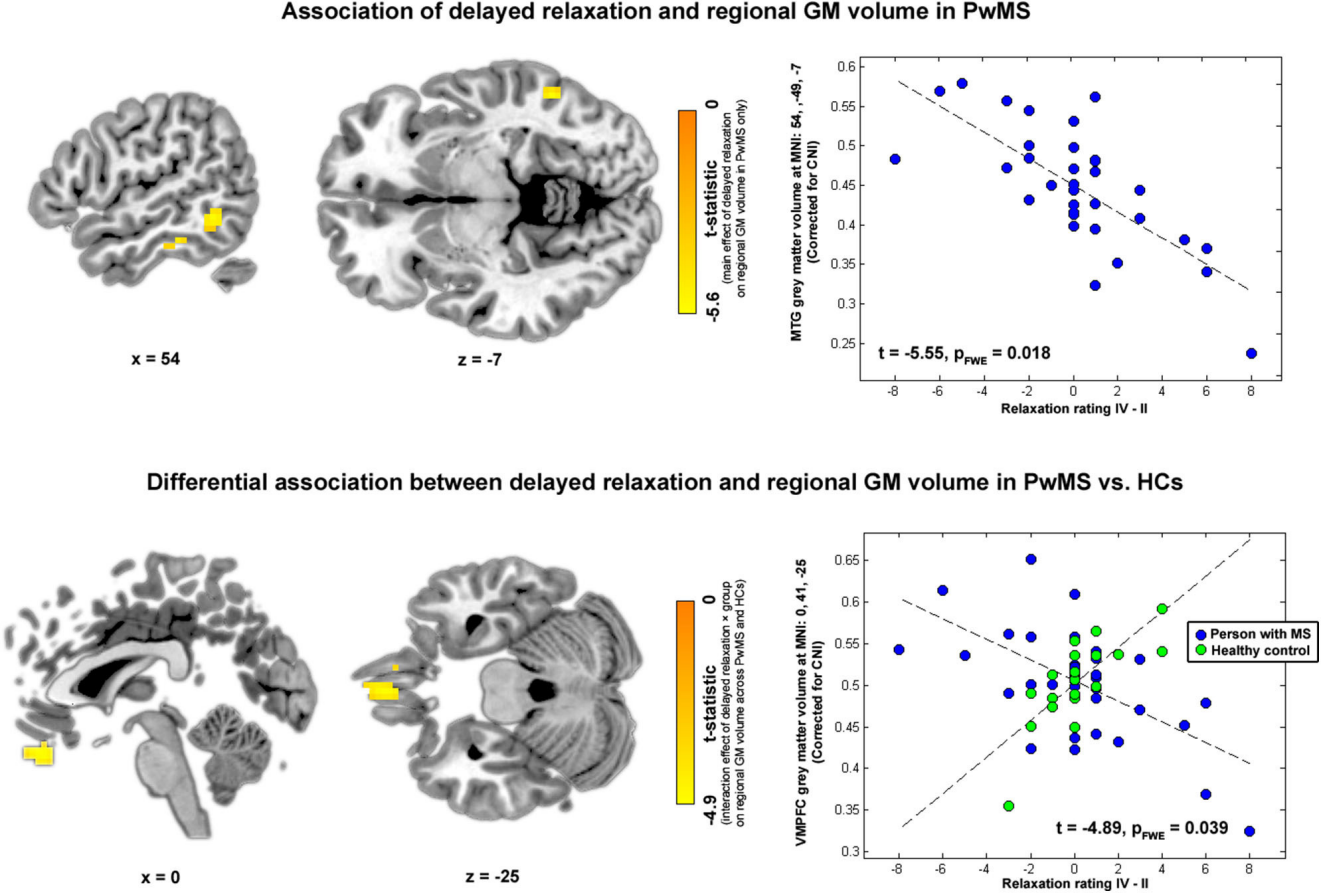
Brain regions and relaxation. Highlighted coordinates in brain slices in the upper panel depict voxels whose GM volume was negatively related to delayed relaxation in PwMS (i.e., showing a negative main effect of delayed relaxation on regional volume in PwMS). The scatterplot on the right side of the upper panel depicts this differential association for the peak voxel coordinate found in the respective analysis, i.e., a coordinate in MTG (MNI: 54, −49, −7). Highlighted coordinates in brain slices in the lower panel depict voxels whose GM volume was differentially related to delayed relaxation in both groups (i.e., voxels showing an interaction effect between delayed relaxation and group on regional volume). The scatterplot on the right side of the lower panel depicts this differential association for the peak voxel coordinate found in the respective analysis, i.e., a coordinate in VMPFC (MNI: 0, 41, −25). Please note that we depict voxels significant according to an uncorrected threshold of α < 10^−3^ for visualization purposes in this figure.

Finally, in the VBM analysis testing interaction effects between relaxation and group on regional GM volume, a negative interaction was found for delayed relaxation and GM volume in ventromedial prefrontal cortex (VMPFC). Please see Table 2 and Figure 4 for further details.

**Table 2:** Brain regions and relaxation.

**Associations of perceived relaxation and regional GM volume in PwMS**						
**MNI region**	**CS**	**x**	**y**	**z**	**t**	**p_FWE_**
***Immediate relaxation***						
FG	27	42	−34	−19	−5.33	0.033
MTG	27	54	−46	−7	−5.17	0.047
***Delayed relaxation***						
MTG	54	54	−49	−7	−5.55	0.018
ENTH	27	24	2	−37	−5.19	0.042
MTG	27	57	−28	−19	−5.13	0.047
**Differential association of perceived relaxation and regional GM volume in PwMS and HCs**						
***Delayed relaxation × group***						
VMPFC	108	0	41	−25	−4.89	0.039

*MNI, Montreal Neurological Institute; CS, cluster size (mm^3^); x, y, z, coordinates in MNI space; t, t statistic; p_FWE_, family-wise-error corrected (α_FWE_ = 0.05) probability for observing the given t value by chance on the voxel level; FG, fusiform gyrus; MTG, middle temporal gyrus; ENTH, enthorinal cortex; VMPFC, ventromedial prefrontal cortex*.

## Discussion

In this study, we investigated psychophysiological and brain morphometric parameters of relaxation in 36 PwMS and related them to those observed in 21 HCs. The main results revealed by our analyses were (1) a different link between relaxation and stress-induced heart rate accelerations in PwMS than HCs, (2) an important role of temporal areas for relaxation in PwMS, and finally (3) a different link between relaxation and regional volume of VMPFC in PwMS than HCs. The VMPFC is a key area of the brain reward system heavily involved in the valuation of affectively relevant stimuli [e.g., (29–31)].

Several analyses were conducted in the study. To evaluate the basic characteristics of our task-based assessment technique and measures, a first analysis of psychophysiological data tested the ability of the task to induce perceived stress and variations in heart rate as well as whether the relaxation parameters were sensitive to the task. Not surprisingly, given the results of a corresponding analysis in (11), the task increased the level of perceived immediate stress and triggered immediate heart rate accelerations across all participants independent of group membership. Exceeding what has been shown in the latter study, the analysis of perceived relaxation data showed that the task induced a significant reduction in feeling relaxed from Rating II to III. Interestingly, no group differences were found in any of the three tested variables—neither for immediate nor for delayed effects. Thus, the overall ability to perceive relaxation and stress appears to be unaffected by MS. Furthermore, none of the measures had a task stage–specific effect that differed between groups/no interaction effects were found. Behavioral analyses relating immediate and delayed stress responses to immediate and delayed relaxation responses showed a negative link in both cases. Importantly, however, although these negative associations were significant, they were not very strong (i.e., 8% mutually explained variation in the markers for immediate effects and 4% for markers of delayed effects). Consequently, one might assume that the relaxation and stress parameters do not reflect the same phenomenon and are thus not redundant. Finally, an analysis relating the immediate to delayed relaxation markers revealed a strong positive relation between the two (47% explained variance). Thus, immediate and delayed relaxation responses appear to emerge from one comparably homogenous latent factor “relaxation after exposure to stress.”

Analyses evaluating associations between relaxation, group, and heart rate revealed that the association between immediate relaxation and immediate heart rate accelerations was significantly more negative in HCs than in PwMS. Specifically, in patients, the link between heart rate accelerations appeared to be very weak, whereas HCs' relaxation ratings appeared to decline together with immediate heart rate accelerations. This finding fits well to results of a study by (16), who investigated decision-making capabilities in PwMS. Specifically, using a gambling task requiring (1) to learn which two of four decks of cards were coupled to a loss across a series of choices and (2) the acquisition and perception of emotional arousal (heightened skin conductance responses; SCRs) before potentially disadvantageous choices (16) found that arousal and performance were lower in PwMS than HCs. Consequently, they concluded that PwMS are characterized by an “impaired emotional reactivity”-–or in other words a reduced ability to perceive interoceptive cues signaling threat. This is strongly related to our previously mentioned finding: Contrary to HCs, PwMS were not able to rest their relaxation assessment on peripheral signals indicating socially evaluative threat.

When the clinical relevance of our relaxation markers was explored by relating them to participants' weak to moderate depressive symptoms, a *positive* link to immediate relaxation was found across all 57 participants (i.e., higher ratings of relaxation were associated with higher BDI-II scores; *t* = 1.99; *p* = 0.045, see Table 1B). Despite not significant on a false positive rate of α = 0.05, the results obtained in the same analysis for the interaction effect (immediate relaxation × group: *t* = 1.62, *p* = 0.108; Table 1B and Figure 3) might suggest that the positive main effect is primarily driven by PwMS. This positive association might become understandable when additionally considering the findings obtained in our second main VBM analysis. Therefore, we would like to point the reader to the Discussion of this second VBM analysis in this regard.

To investigate brain volumetric correlates of relaxation, we conducted two main and four supplementary VBM analysis. In the first main analysis, associations between relaxation on regional GM volume in PwMS only were tested. Results of this analysis suggest an important role of temporal areas for relaxation as regional volumes in FG and MTG (MTG and ENTH) were negatively linked to immediate (delayed) relaxation. Interestingly, given the close link between relaxation and resilience, these temporal areas are involved in threat processing (32) and among key areas found in PTSD studies. In particular (33) found a volume reduction in PTSD patients compared with HCs in MTG and FG. Moreover (34) revealed a negative link between the frequency of re-experiencing symptoms and GM volume in MTG in PTSD and (35) a positive association between resilience assessed with a questionnaire and GM thickness in FG of HCs. Finally, persons confronted with traumatizing events who did not develop a PTSD in response to trauma exposure had a higher cortical thickness in FG than persons developing PTSD (36). Consequently, the regions identified as being related to relaxation in PwMS match those found in related studies on resilience and PTSD.

However, despite the fact that the regions found in our study strongly overlap with regions found in PTSD studies, the negative direction of associations between relaxation and temporal volumes appears as counter-intuitive as the positive link between task-based relaxation and depressive symptoms reported (primarily for PwMS) earlier. This becomes evident when considering that the volume of temporal areas was *positively* linked to resilience in HCs (35) or that the frequency of re-experiencing PTSD symptoms and GM volume in MTG was negatively associated (34). Consequently, one might ask whether the neural mechanism underlying the relaxation evaluation is biased in PwMS.

The results of the second main VBM analysis, which revealed a differential coupling between relaxation and regional GM volume in PwMS and HCs for VMPFC coordinates together with the finding of a differential coupling of relaxation and heart rate in PwMS and HCs, argue that this is presumably the case. This becomes clear when the functional roles of VMPFC are considered. Specifically, the VMPFC is a key area of the brain reward system that links the value of stimuli [positive for rewarding ones such as food [e.g., (30)], negative for punishing ones such as electric shocks [e.g., (37)] to hedonic experience (31). Within this framework, activity of this area can be considered as a neuronal safety/threat signal as this area responds stronger to reward (-predictive) than to punishment (-predictive) stimuli in healthy persons. Moreover, because VMPFC is also a regulator of sympathetic nervous functions (38), it also triggers a peripheral safety/threat signal of opposing direction as the neural one in healthy individuals (e.g., an increase in SCRs in response to punishment-predictive stimuli). Moreover, both behaviors change under threat in the healthy: VMPFC activity decreases and SCRs increase (37).

These responses, however, are altered in PwMS and this alteration has behavioral consequences: In line with our earlier assumption of a biased processing of affectively relevant, reward-related stimuli in MS, SCRs to punishment-predictive stimuli were weaker in PwMS than in HCs in the gambling task used by (16) and their task performance (relying on the perception of these punishment-predictive responses) was worse. Simultaneously, the worse performance of PwMS in this task is related to VMPFC activity as our recent fMRI study employing the same task as (16) in the participants that were also analyzed in the present study showed that VMPFC activity is positively associated with task performance across PwMS and HCs despite this difference in performance (20).

Given the existing work on stress-related processes contributing to GM alterations in MS, conceiving a mechanism underlying this altered link between relaxation and regional VMPFC volume in MS is a challenging task. Speculatively, a candidate mechanism might be inferred from (1) chronic glucocorticoid system/HPA hyperactivity and thus insensitivity repeatedly observed (in certain tissues) in PwMS [e.g., (6)] and (2) the fact that glucocorticoid insensitivity of the rat VMPFC alters stress-processing or more specifically induces a hypersensitivity to stress indicated by, e.g., depression-like behavior (39). Consequently, based on the existing literature, one might speculate that glucocorticoid insensitivity of the VMPFC due to HPA hyperactivity in MS is accompanied by a systematic misinterpretation of stress signals which might in turn be related to the extent of depressive symptoms and thus clinically relevant. Moreover, given the previously shown link between HPA hyperactivity and hippocampal atrophy in more depressed PwMS (9) and the overall role of hippocampal and neighboring enthorinal areas in mood regulation (40), one might further speculate that this or a related mechanism might also drive the negative link between relaxation and regional temporal GM volume in PwMS. Please note that this explanation might hold despite the fact that PwMS were not characterized by less VMPFC or temporal volume (at these specific coordinates) than HCs as the MRI technique applied in the present study (i.e., evaluating MP-RAGE images with VBM) might miss variations in the tissue microstructure [such as a reduced glucocorticoid receptor density underlying stress hypersensitivity in (39); cf. (41)].

In addition to the two VBM analyses presented in the main text, four supplementary VBM analyses were conducted. The first tested volume differences between PwMS and HCs and identified loss of GM volume in PwMS in thalamic areas (which is consistent with findings on MS-induced atrophy) and, to a lesser degree, in MTG (42, 43). However, these areas characterized by GM volume loss in PwMS turned out to be of limited importance for relaxation-related tissue alterations in PwMS because a repetition of the two VBM analyses presented in the main text constrained to the coordinates found in the first supplementary VBM analysis in supplementary VBM analysis 2 did not reveal any significant associations. When additionally considering that non-atrophy affected areas did show an association with relaxation in PwMS, this finding might underline the possibility of subtle, functionally relevant, MS-driven tissue alterations which are within the range of tissue variations observed in the healthy.

To facilitate a comparison between associations of relaxation and regional GM volume in PwMS with those found in HCs, we investigated associations of relaxation and regional GM volume in HCs only in supplementary VBM analysis 3. Using a sensitive significance threshold (α_uncorrected_ = 0.001), this analysis revealed several coordinates with positive and negative associations primarily located in temporal, visual, and parietal regions. None of these areas reached significance according to the more conservative threshold applied in the corresponding PwMS VBM analysis (i.e., α_FWE_ = 0.05). However (within this more liberal testing framework), supplementary VBM analysis 3 obtained results compatible with those reported in the resilience and PTSD literature mentioned earlier (34, 35), i.e., that regional volume of several FG and inferior temporal gyrus coordinates was positively related to immediate and delayed relaxation in HCs. Moreover, none of the temporal coordinates with a negative association in PwMS did spatially overlap with any of the coordinates found in HCs—neither with those showing a positive, nor with those showing a negative association with immediate or delayed relaxation in HCs. Consequently, given the similarity with findings obtained in resilience and PTSD studies, the results of this supplementary analysis argue that the somewhat counter-intuitive negative association between temporal areas in GM regions and relaxation in PwMS is a valid finding not driven by measurement error. Moreover, given the missing spatial overlap between coordinates found in the more sensitive supplementary HC analysis and the coordinates with negative associations found in the more conservative PwMS analysis, one might assume that the negative associations found in PwMS are driven by a disease-specific process.

Finally, in supplementary VBM analysis 4, we evaluated whether the association between regional GM volume variations and relaxation in PwMS found in the first VBM analysis presented in the main text was driven by the MS disease progression by repeating the first main analysis with an additional covariate of no interest that reflected whether a given patient was a relapsing-remitting or a secondary progressive MS patient. Given the very strong overlap between results obtained in the main and the supplementary VBM analysis, one can conclude that the impact of a progressive disease type on the association between perceived relaxation and regional GM volume in PwMS is negligible.

A limitation of the study is the fact that although there are findings in the literature allowing to speculate on how regional GM variations might lead to a different link between relaxation and regional volume in VMPFC or temporal areas in PwMS than HC, the explanations derived from these findings remain speculative. Consequently, the investigation of more specific mechanisms underlying these associations incorporating additional MS-related neurobiological parameters such as T-cell glucocorticoid (in-) sensitivity [cf. (6, 44)] remains a target of future studies.

Taken together, we found a differential coupling between relaxation and regional VMPFC volume in PwMS and HCs as well as a differential coupling of relaxation and stress-related heart rate accelerations in PwMS and HCs that appear to reflect a systematic misinterpretation of stress signals in MS.

## Data Availability Statement

The raw data supporting the conclusions of this article will be made available by the authors, without undue reservation.

## Ethics Statement

The studies involving human participants were reviewed and approved by Research ethics committee of the Charité–Universitätsmedizin Berlin (EA1/182/10, amendment V). The patients/participants provided their written informed consent to participate in this study.

## Author Contributions

KW: conception, investigation, writing original draft, and writing review. FE, KR, TS-H, and AB: writing original and writing review. SH: writing original, writing review, and software. JB-S: writing original, writing review, and investigation. J-DH: writing original, writing review, and funding acquisition. SG: writing original, writing review, and conception. FP: writing original, writing review, conception, funding acquisition, and project administration. MW: conceptualization, formal analysis, investigation, methodology, project administration, software, supervision, validation, visualization, writing—original draft, writing—review and editing. All authors contributed to the article and approved the submitted version.

## Conflict of Interest

The authors declare that the research was conducted in the absence of any commercial or financial relationships that could be construed as a potential conflict of interest.
