# Supplementary material for: Altered Coupling of Psychological Relaxation and Regional Volume of Brain Reward Areas in Multiple Sclerosis
Source: Front Neurol. 2020 Oct 6;11:568850. doi: 10.3389/fneur.2020.568850 (PMC7574404; doi:10.3389/fneur.2020.568850)
Supplement: Supplementary file 1 [file Presentation_1.pdf]

Altered coupling of psychological relaxation and regional volume of brain reward areas in multiple sclerosis

Katharina Wakonig[1], Fabian Eitel[2,3], Kerstin Ritter[2,3], Stefan Hetzer[3], Tanja Schmitz-Hübsch[1,4], Judith Bellmann-Strobl[1,4], John-Dylan Haynes[1,3,5], Alexander U. Brandt[1,6], Stefan M. Gold[7,8,9*], Friedemann Paul[1,4,10*], Martin Weygandt[1,4*,§]

[1] Charité – Universitätsmedizin Berlin, corporate member of Freie Universität Berlin, Humboldt-Universität zu Berlin, and Berlin Institute of Health, NeuroCure Clinical Research Center, 10117 Berlin, Germany.
[2] Charité – Universitätsmedizin Berlin, corporate member of Freie Universität Berlin, Humboldt-Universität zu Berlin, Berlin Institute of Health (BIH), Department of Psychiatry and Psychotherapy, 10117 Berlin, Germany
[3] Charité – Universitätsmedizin Berlin, corporate member of Freie Universität Berlin, Humboldt-Universität zu Berlin, and Berlin Institute of Health, Berlin Center for Advanced Neuroimaging, 10117 Berlin, Germany.
[4] Max Delbrück Center for Molecular Medicine and Charité – Universitätsmedizin Berlin, corporate member of Freie Universität Berlin, Humboldt-Universität zu Berlin, and Berlin Institute of Health, Experimental and Clinical Research Center, 13125 Berlin, Germany.
[5] Charité – Universitätsmedizin Berlin, corporate Member of Freie Universität Berlin, Humboldt-Universität zu Berlin, Berlin Institute of Health, Bernstein Center for Computational Neuroscience, 10117, Berlin, Germany.
[6] Department of Neurology, University of California, Irvine, CA, USA
[7] Institute of Neuroimmunology and Multiple Sclerosis (INIMS), University Medical Center Hamburg-Eppendorf, 20251 Hamburg, Germany.
[8] Charité – Universitätsmedizin Berlin, corporate member of Freie Universität Berlin, Humboldt-Universität zu Berlin, and Berlin Institute of Health, Department of Psychiatry and Psychotherapy, Campus Benjamin Franklin, 12203 Berlin, Germany.
[9] Charité – Universitätsmedizin Berlin, corporate member of Freie Universität Berlin, Humboldt-Universität zu Berlin, and Berlin Institute of Health, Department of Psychosomatic Medicine, 10117 Berlin, Germany.
[10] Charité – Universitätsmedizin Berlin, corporate member of Freie Universität Berlin, Humboldt-Universität zu Berlin, and Berlin Institute of Health, Department of Neurology, 10117 Berlin, Germany

Supplementary material

[*] These authors contributed equally to this work.

[§] Corresponding author. Charité – Universitätsmedizin Berlin, Chariteplatz 1, 10117 Berlin. Email: martin.weygandt@charite.de.

Methods

Statistical analyses of MRI group data

Supplementary VBM analysis 1: Analysis of differences in regional GM volume between PwMS and HCs

In order to identify areas with smaller regional GM volume in PwMS than HCs, we conducted a regression analysis using SnPM13. Group membership (0: HC, 1, PwMS) served as CI, sex, age, the participants intracranial volume and a constant served as CNI. Exactly as all other VBM MRI analyses, this analysis was constrained to coordinates located in the GM group mask. In this explorative analysis, we report the MNI-coordinates for the voxel with most extreme t-statistic (i.e. for the peak voxel) in a cluster of voxels reaching significance following a multiple comparison or family-wise error (FWE) corrected significance threshold (α_{FWE} = 0.1) for negative effects.

Supplementary VBM analysis 2: Anatomically constrained analyses of associations of perceived relaxation and regional GM volume in PwMS and of differential association of perceived relaxation and regional GM volume in PwMS and HCs

In the second supplementary VBM analysis, we repeated the two VBM analyses described in the main text. The only difference between the analyses presented in the main text and this supplementary one was, that we exclusively searched across voxel coordinates identified as significantly affected by GM loss in PwMS in the above supplementary analysis in the present supplementary analyses.

Supplementary VBM analysis 3: Analysis of associations of perceived relaxation and regional GM volume in HCs

In this supplementary analysis, we tested associations between regional GM volume and immediate and delayed relaxation in the 21 HCs. Except for the uncorrected false positive threshold used for inference ($\alpha = 0.001$), all aspects were as described in the main text for the corresponding analysis in PwMS. Given that the primary aim of this analysis was to enable a comparison of main effects of relaxation on regional GM volume in HCs to those observed in PwMS, the more liberal uncorrected significance threshold of $\alpha = 0.001$ was chosen to compensate for the loss of statistical power in the HC analysis following from the smaller number of HCs compared to PwMS.

In a follow-up step, we evaluated whether the coordinates found in the first VBM analysis presented in the main text in PwMS (exclusively showing negative associations between GM volume and relaxation) did spatially overlap with any of the coordinates found in supplementary VBM analysis 3 (irrespective of the directionality of associations found in the supplementary analysis and the type of relaxation [i.e., immediate and delayed]).

Supplementary VBM analysis 4: Analysis of associations between relaxation and regional GM volume in PwMS additionally considering the putative impact of progressive MS

To test whether the presence of (secondary) progressive MS (vs. RRMS) has an impact on the link between perceived relaxation and regional GM volume in MS, we repeated the corresponding analyses presented in the main text but now included a regressor coding ones for SPMS and zeros for RRMS as additional CNI. This was done separately for immediate and for delayed relaxation. Due to the inclusion of an additional CNI, we report the MNI-coordinates for the voxel with most extreme t-statistic (i.e. for the peak voxel) in a cluster of voxels reaching significance following a multiple comparison or family-wise error (FWE) corrected significance threshold of $\alpha_{FWE} = 0.1$.

Results

Statistical analyses of MRI group data

Supplementary VBM analysis 1: Analysis of differences in regional GM volume between PwMS and HCs

The below supplementary Table S1 reports coordinates with significant less GM volume in PwMS than HCs.

Table S1. Brain regions with less GM volume in PwMS than HCs. Abbreviations: MNI: Montreal Neurological Institute; CS: cluster size in mm^3; x, y, z: coordinates in MNI space; t: t–statistic; $p_{FWE:}$ family-wise-error corrected ($\alpha_{FWE} = 0.1$) probability for observing the given t-value by chance on the voxel level.

MNI region	CS	x	y	z	t	p_{FWE}
Thalamus (r)	999	12	-34	5	-5.03	0.021
Middle temporal gyrus (l)	513	-57	-61	8	-4.86	0.035
Planum temporale (r)	81	51	-25	11	-4.76	0.046
Middle occipital gyrus (l)	54	-42	-82	29	-4.59	0.068
Precentral gyrus (r)	27	9	-22	47	-4.47	0.091
Cerebellum (l)	27	-18	-34	-28	-4.47	0.093
Inferior temporal gyrus (l)	27	-63	-46	-19	-4.46	0.096

Supplementary VBM analysis 2: Anatomically constrained analyses of associations of perceived relaxation and regional GM volume in PwMS and of differential association of perceived relaxation and regional GM volume in PwMS and HCs

These supplementary analyses testing associations of perceived relaxation and regional GM volume in PwMS and differential association of perceived relaxation and regional GM volume in PwMS and HCs in brain areas significantly affected by GM loss in PwMS did not reveal any significant results.

Supplementary VBM analysis 3: Analysis of associations of perceived relaxation and regional GM volume in HCs

The below supplementary tables report GM coordinates positively or negatively related to regional GM volume in HCs in terms of immediate relaxation (Table S2) or delayed relaxation respectively (Table S3).

Table S2. Brain regions and immediate relaxation in HCs. Abbreviations: MNI: Montreal Neurological Institute; CS: cluster size in mm^3; x, y, z: coordinates in MNI space; t: t–statistic; $p_{uncorr.}$: uncorrected probability for observing the given t-value by chance on the voxel level (α = 0.001). p_{FWE}: family-wise-error corrected probability for observing the given t-value by chance on the voxel level. Please note, that the non-monotonic increase in uncorrected p-values across t-statistics with decreasing absolute magnitude results from the permutation method applied by SnPM13.

**Immediate relaxation**							
**MNI region**	**CS**	**X**	**y**	**z**	**t**	$p_{uncorr.}$	p_{FWE}
Temporal pole	81	-27	17	-43	4.61	0.0004	0.488
Fusiform gyrus	54	39	-10	-31	4.39	0.0002	0.581
Inferior temporal gyrus	108	-57	-10	-34	4.32	0.0006	0.610
Middle frontal gyrus	54	-24	-1	50	4.02	0.0008	0.740
Gyrus rectus	54	3	32	-31	3.97	0.0004	0.763
Temporal pole	27	-33	14	-46	3.86	0.0004	0.800
Temporal pole	27	-30	8	-49	3.42	0.0008	0.926
Lingual gyrus	594	9	-82	-10	-6.03	0.0002	0.105
Calcarine cortex	567	6	-91	5	-5.31	0.0002	0.242
Precentral gyrus	54	-36	-1	47	-4.91	0.0002	0.369
Supramarginal gyrus	81	60	-40	26	-4.62	0.0002	0.473
Supramarginal gyrus	81	-54	-49	26	-4.45	0.0004	0.539
Postcentral gyrus	81	36	-34	53	-4.11	0.0004	0.683
Inferior occipital gyrus	27	-48	-76	-1	-3.95	0.0006	0.746
Lingual gyrus	54	-3	-94	-19	-3.84	0.0006	0.787
Fusiform gyrus	81	-30	-34	-25	-3.82	0.0006	0.794
Supramarginal gyrus	54	-57	-49	35	-3.77	0.0008	0.810
Lingual gyrus	54	9	-70	2	-3.76	0.0008	0.815
Lingual gyrus	27	-6	-67	2	-3.73	0.0008	0.826

Table S3. Brain regions and delayed relaxation in HCs. Abbreviations: MNI: Montreal Neurological Institute; CS: cluster size in mm^3; x, y, z: coordinates in MNI space; t: t–statistic; $p_{uncorr.}$: uncorrected probability for observing the given t-value by chance on the voxel level (α = 0.001). p_{FWE}: family-wise-error corrected probability for observing the given t-value by chance on the voxel level. Please note, that the non-monotonic increase in uncorrected p-values across t-statistics with decreasing absolute magnitude results from the permutation method applied by SnPM13.

Delayed relaxation							
**MNI region**	**CS**	**x**	**y**	**z**	**t**	$p_{uncorr.}$	p_{FWE}
Precentral gyrus	108	-48	5	14	4.71	0.0002	0.418
Cerebellum exterior	108	18	-52	-61	4.18	0.0006	0.646
Cerebellum exterior	81	39	-61	-61	4.09	0.0006	0.691
Fusiform gyrus	162	27	-13	-40	3.84	0.0002	0.790
Inferior temporal gyrus	27	-51	-52	-16	3.61	0.0006	0.867
Middle frontal gyrus	27	39	14	53	3.57	0.0008	0.874
Thalamus proper	81	12	-28	2	-4.44	0.0002	0.566
Superior parietal lobule	27	-33	-55	56	-4.10	0.0008	0.711
Thalamus proper	27	18	-31	5	-3.66	0.0006	0.871

The follow-up step, which evaluated whether the coordinates found in the first VBM analysis presented in the main text in PwMS (exclusively showing negative associations between GM volume and relaxation) did spatially overlap with any of the coordinates found in supplementary VBM analysis 3 showed, that none of the temporal coordinates with a negative association in PwMS spatially overlapped with any of the coordinates found in HCs. Specifically, these coordinates did neither overlap with those showing a positive, nor with those showing a negative association with immediate or delayed relaxation in HCs.

Supplementary VBM analysis 4: Analysis of associations between relaxation and regional GM volume in PwMS additionally considering the putative impact of progressive MS

The below supplementary Table S4 reports coordinates with an association between relaxation measures and regional GM volume in PwMS according to a regression model including MS type (0 – RRMS, 1 – SPMS) as additional CNI.

Table S4. Brain regions related to relaxation in PwMS. Abbreviations: MNI: Montreal Neurological Institute; CS: cluster size in mm^3; x, y, z: coordinates in MNI space; t: t–statistic; $p_{FWE:}$ family-wise-error corrected (α_{FWE} = 0.1) probability for observing the given t-value by chance on the voxel level. SFG, superior frontal gyrus; TPL, temporal pole.

Predictor	MNI region	CS	x	y	z	t	p_{FWE}
**Relaxation (immediate)**	FG	27	42	-34	-19	-5.15	0.049
	MTG	27	54	-46	-7	-4.99	0.065
**Relaxation (delayed)**	MTG	108	54	-49	-7	-5.39	0.033
	ENTH	81	24	2	-37	-5.14	0.051
	MTG	27	57	-28	-19	-4.98	0.070
	SFG	27	18	38	41	-4.90	0.083
	TPL	27	24	11	-40	-4.81	0.098
